# Clinical significance of miR-9-5p in NSCLC and its relationship with smoking

**DOI:** 10.3389/fonc.2024.1376502

**Published:** 2024-04-02

**Authors:** Tian-Xiang Zhang, Xin-Chun Duan, Yong Cui, Ye Zhang, Meng Gu, Zi-Yu Wang, Wei-Ying Li

**Affiliations:** ^1^Department of Thoracic Surgery, Beijing Friendship Hospital, Capital Medical University, Beijing, China; ^2^Department of Infectious Medicine, Beijing Friendship Hospital, Capital Medical University, Beijing, China; ^3^Cancer Research Center, Beijing Chest Hospital, Capital Medical University/Beijing Tuberculosis and Thoracic Tumor Research Institute, Beijing, China

**Keywords:** miR-9-5p, non-small-cell lung cancer, smoking, proliferation, migration

## Abstract

**Purpose:**

Dysregulated expression of microRNA (miRNAs) in lung cancer has been wildly reported. The clinicopathologic significance of miR-9-5p in non-small-cell lung cancer (NSCLC) patients and its effect on NSCLC progression were explored in this study.

**Patients and methods:**

A total of 76 NSCLC patients were included. miR-9-5p expression was evaluated by real-time quantitative polymerase chain reaction (RT-qPCR). Then, *in vitro* experiments including cell growth curve assays, colony formation assays, and transwell migration assays were performed. Further clinicopathological and prognostic values were explored using bioinformatics analysis of the TCGA database.

**Results:**

miR-9-5p expression was significantly increased in tumor tissues (both *P* < 0.0001). miR-9-5p expression was relatively higher in larger tumors (*P* = 0.0327) and in lung squamous carcinoma (LUSC) (*P* = 0. 0143). In addition, miR-9-5p was significantly upregulated in the normal lung tissues of cigarette smokers (*P* = 0.0099). *In vitro*, miR-9-5p was correlated with cell proliferation and migration. After that, bioinformatics analysis of the TCGA database indicated that miR-9-5p was correlated with tumor size (*P* = 0.0022), lymphatic metastasis (*P* = 0.0141), LUSC (*P* < 0.0001), and smoking history (*P* < 0.0001). Finally, a prognostic study indicated high miR-9-5p expression was correlated with poor prognosis in LUAD (*P* = 0.0121).

**Conclusion:**

Upregulation of miR-9-5p may have an oncogenic effect in NSCLC and may be related to smoking. The conclusion of this study may help find new prognostic and therapeutic targets for NSCLC and the exploration of the relationship between smoking and lung cancer.

## Highlights

- Significant findings: The upregulation of miR-9-5p may play an oncogenic role in NSCLC and may be related to smoking.
- Value of the study: miR-9-5p was found to be highly expressed in NSCLC tumor tissues, especially in LUSC, and is associated with malignant characteristics of NSCLC. Its oncogenic effect was confirmed *in vitro*. Furthermore, increased expression of miR-9-5p is closely related to smoking.

## Introduction

Lung cancer leads to numerous cases of cancer-related mortality worldwide, and it is one of the most common neoplasms at present (1). Among these, most lung cancers are NSCLCs. However, the recurrence rate of NSCLC in patients remains at a high level, with a poor 5-year overall survival even in the early stages, despite the development in diagnostic and therapeutic methods (2). In order to improve prognoses for patients with NSCLC, we must strive for the further development of diagnosis and therapy. Besides, a better understanding of the pathological mechanism of NSCLC at the genetic level is also necessary. Therefore, more attention should be given to the exploration of genetic and oncogenesis characteristics of NSCLC to find a specific gene target that influences the current diagnostics. Abnormal expression of miRNAs that are associated with cancer has been extensively investigated by many studies before, and the diagnostic or prognostic significance of miRNA has also been reported frequently (3). In cancer cells, miRNA has been reported to exhibit both promotive and suppressive roles in cancer progression (4). Furthermore, cigarette smoking can also lead to changes in lung miRNA expression, and the alteration of certain miRNA expressions caused by smoking may be potentially related to the occurrence and development of tumors (5, 6). In the past, Holownia et al. found that cigarette smoke intervention could reduce the level of miR-9 in A549 cell line *in vitro*, but their research lacked clinical evidence to support this finding (7). In addition, few studies have focused on the correlation between miR-9 and smoking.

miR−9, including miR−9−1, miR-9-2 and miR-9-3, is encoded by three chromosomal loci distributed in chromosome 1q22, 5q14.3, and 15q26.1 respectively (8). Interestingly, the study results about miR-9 in NSCLC are controversial. It has been reported to have an oncogenic effect in NSCLC; previous studies shown that miR-9 promotes proliferation, migration, and invasion ability by targeting tumor suppressor genes (9–12). Meanwhile, a tumor-suppressive effect that could inhibit the malignant phenotype of NSCLC cell has also been reported (13–15). Besides, overexpression of miR-9 may enhance the radiosensitivity (14, 16) and cisplatin sensitivity (17). However, previous studies on the effect of miR-9-5p in NSCLC patients were limited and lacking rigorous *in vitro* experimental verification. Besides, few studies focus on the correlation between miR-9-5p and cigarette smoking.

Herein, we focused on the clinicopathological significance of miR-9-5p in NSCLC. The promotion effect on proliferation and migration was also verified using *in vitro* cell phenotypic experiments, and the prognostic value of miR-9-5p was explored by bioinformatics analysis. In addition, the relationship between miR-9-5p and cigarette smoking was explored preliminarily.

## Patients and methods

### Patients

Between 2018–10 and 2020-1, 76 NSCLC patients hospitalized in the Deportment of Thoracic Surgery of Beijing Chest Hospital were enrolled. Their ages ranged from 26 to 79 years old (mean = 61.67). After pathological diagnosis, the fresh tumor tissues and corresponding adjacent noncancerous lung tissues of each enrolled patients were collected. Any patients receiving chemotherapy or radiation therapy before surgery and/or those with any other accompanying malignant disease were excluded. The tissue samples were placed into liquid nitrogen for at least 30 minutes within 2 hours after resection and then transferred to a fridge at a temperature of −80°C for long-term storage. The clinical significance of miR-9-5p and the involved clinicopathological factors, such as age, sex, LUSC or LUAD, smoking history, greatest tumor diameter, pleura invasion, lymphatic metastasis, and vessel carcinoma embolus, were estimated. Written informed consent was provided by all participants, and the Ethics Committee of Beijing Chest Hospital approved the establishment and performance of this study (No. 2019-71). The information of included participants and the clinicopathological analysis results are listed in **Table 1**.

**Table 1:** Clinicopathological parameter of participants and miR-9-5p expression in malignant and non-malignant tissues.

Variables	No.	Tumor tissues (Median, 25–75 Percentile)	P value	Nonmalignant tissues (Median, 25–75 Percentile)	P value
Ages			0.1407		0.9313
≤65	49	0.5763, 0.1130-3.360		0.07925, 0.03638-0.1883	
>65	27	0.5195, 0.1765-2.568		0.08768, 0.0552-0.1578	
Sex			0.1799		0.0430*
Female	28	0.5115, 0.06375-1.327		0.06831, 0.0307-0.135	
Male	48	0.6384, 0.1721-3.363		0.09384, 0.06227-0.2016	
Histologic type			0.0143*		0.2342
LUAD	51	0.4890, 0.1063-1.514		0.07703, 0.03762-0.1443	
LUSC	25	1.589, 0.2000-4.135		0.1006, 0.06037-0.2005	
Smoking or not			0.6368		0.0099**
Non-smoker	38	1.589, 0.2000-4.135		0.06891, 0.02984-0.1239	
Smoker	37	0.5195, 0.1627-3.360		0.1006, 0.06735-0.2089	
Tumor size			0.0327*		0.0465*
≤2 cm	18	0.1684, 0.03674-1.831		0.03807, 0.01458-0.1954	
>2 cm	58	0.6610, 0.1886-3.333		0.08838, 0.06108-0.1653	
Lymphatic metastasis			0.5718		0.6360
Negative	50	0.4954, 0.1494-2.765		0.08235, 0.03744-0.1912	
Positive	26	0.6213, 0.1481-3.346		0.08748, 0.05741-0.1607	
Pleura invasion			0.2407		0.1036
Negative	45	0.3473, 0.1149-1.790		0.0738, 0.03726-0.1363	
Positive	26	0.7088, 0.1366-3.346		0.0992, 0.07007-0.1721	
Vessel carcinoma embolus			0.3894		0.5628
Negative	46	0.3573, 0.1564-2.154		0.08037, 0.03663-0.1912	
Positive	29	0.7008, 0.1065-3.525		0.08908, 0.06549-0.1511	

*Statistically significant, P < 0.05; **Statistically significant, P < 0.01.

### Cell culture and transfection

Eight Human NSCLC cell lines and one pulmonary epithelial cell line (BEAS-2B) were selected for primary screening in this study. Among them, EKVX, H522, H23, and H226 were from the National Institutes of Health (NIH). H1703, H1299, H1395, A549, and BEAS-2B were acquired from the National Infrastructure of Cell Line Resource (NICR). RPMI-1640 medium (Invitrogen, Carlsbad, USA) with 10% fetal bovine serum (FBS; Gibco, Los Angeles, USA) was used for cell culture. The cells were incubated at 37° C with 5% CO_2_ in a constant temperature incubator.

The miR-9-5p overexpression (miR-9-5p-OE) plasmid, miR-9-5p inhibition (miR-9-5p-KD) plasmid, and corresponding negative control plasmid were purchased from Shanghai Genechem Co., Ltd (Shanghai, China). According to the RT-qPCR results of NSCLC cell lines, the miR-9-5p-OE plasmids were transfected into A549 cells and miR-9-5p-KD plasmids were transfected into H1299 cells; the corresponding empty vector was also transected as a negative control. The transfection process was performed using Lipofectamine™ 3000 according to the manufacturer’s guidelines (Thermo Fisher Scientific, USA). In order to establish the stable cell lines, transfected A549 and H1299 cells were selected with 600 mg/ml G418 (Sigma-Aldrich, St. Louis, MO, USA).

### RT-qPCR of miR-9-5p

The mirVana™ miRNA Isolation Kit (Thermo Fisher Scientific, Vilnius, LTU) was selected for total RNA isolation of tissue samples and cells following the instruction.

Then, we synthesized cDNA using a TaqMan™ MicroRNA Reverse Transcription Kit (Thermo Fisher Scientific, Vilnius, LTU) from total RNA isolated previously. Afterwards, we performed RT-qPCR using the following protocol: 50°C for 2 min, then heated up to 95°C and maintained for 10 min, followed by 40 cycles of circulation at 95°C for 15 sec and 60°C for 60 sec. The RT-qPCR procedure was carried out using Applied Biosystems 7500 (Thermo Fisher Scientific, Waltham, MA, USA) and TaqMan^®^ MicroRNA Assays (Thermo Fisher Scientific, Pleasanton, CA, USA). We selected U6 small nuclear RNA (U6) as an endogenous control using the 2^−ΔΔCt^ method for RT-qPCR data normalizing. A549 was chosen as a reference sample for tissue samples, while BEAS-2B was chosen as a reference sample for all NSCLC cell lines. A reference sample was used in each reaction, and every measurement was performed for three replicates.

MicroRNA-specific stem-loop RT primers (Thermo Fisher Scientific, Pleasanton, CA, USA) assay ID: hsa-miR-9-5p 000583, U6 snRNA 001973.

MicroRNA-specific TaqMan™ MGB probes (Thermo Fisher Scientific, Pleasanton, CA, USA) assay ID: hsa-miR-9-5p 000583, U6 snRNA 001973.

We selected U6 small nuclear RNA (U6) as an endogenous control using the 2^−ΔΔCt^ method for RT-qPCR data normalizing. A reference sample was used in each reaction, and every measurement was performed for three replicates.

### Cell growth curve assay

The proliferation ability of selected cell lines was examined using a Cell Counting Kit-8 (CCK-8; Dojindo Molecular Technologies, Kumamoto, Japan) assay according to the instruction. Transfected A549 and H1299 and corresponding negative controls were seeded into flatbottom 96-well plates. A total of 4000 cells per well for A549 and 5000 cells per well for H1299 were used. After seeding, 96-well plates were maintained in constant temperature incubator for 24, 48, 72, and 96 hours respectively. Then, we added CCK-8 reagent into 96-well plates at a volume of 10 μL per well. After that, the 96-well plates were incubated at 37°C in a constant temperature incubator with 5% CO_2_ for 2 hours. At last, we used the EL-800 Universal Microplate Reader (BioTek Instruments, Inc., Winooski, VT, USA) to measure the absorbance values of each well at 450 nm.

### Colony formation assay

We used 2 mL culture medium to suspend the cells, seeding the cells into six-well plates. Approximately 200 A549 cells or 300 H1299 cells per wells were seeded. After seeding, cells were incubated at 37°C with 5% CO_2_ for 7 to 14 days depending on cell types. After the incubation was halted, we washed the cells twice with PBS, using 2% crystal violet for fixation and staining. We used an optical microscope to count the colonies that had more than 50 cells.

### Transwell migration assay

A transwell migration assay was performed using an 8μm pore polycarbonate membrane 24-well transwell unit (Corning Incorporated, Costar, USA, ref 3422). The transwell unit was treated with Basement Membrane (Corning Incorporated, Matrigel Matrix, USA, ref 356234) preliminarily according to instruction. filling the lower compartment with 10% FBS medium as a chemoattractant. In the meantime, after overnight starvation, resuspended cells were plated in the upper compartment with serum-free medium. After incubation for 24 hours at 37°C with 5% CO_2_, the cells in the upper compartment were completely wiped off by gentle swabbing. We used crystal violet to stain the cells that had migrated to the lower surface of the membranes for 10 minutes. Then, we took photos and counted invading cells (using Image J to calculated) in five 200× magnifying fields.

### Bioinformatic analyses in TCGA

Bioinformatic analysis of TCGA data was performed in order to validate the clinicopathological significance ulteriorly and the prognostic value of miR-9-5p. miR-9-5p expression data and available clinical information were downloaded using Assistant for Clinical Bioinformatics (https://www.aclbi.com/) and OncoLnc (18)(http://www.oncolnc.org/). As for prognostic analysis, univariate analysis was performed using Kaplan-Meier survival analysis, and multivariate analysis was performed using Cox regression analysis. Beyond that, high-throughput sequencing data of LUAD and LUSC patients were downloaded from UCSC XENA (https://xenabrowser.net/), including gene expression data, clinical phenotype, and miRNA expression quantification data. This part of the statistical analysis was completed in R 4.2.0. The proportion of infiltrated immune cells of tumor tissues in different subgroups was compared using the CYBERSORT algorithm (IOBR package). Potential targets of miR-9-5p were found by miRDB (https://mirdb.org/); KEGG and GO enrichment analysis of those genes was performed using clusterProfiler.

### Statistical analysis

SPSS version 22.0 (Chicago, IL, USA) and GraphPad Prism version 9.5 (La Jolla, CA, USA) were selected for statistical analysis and preparing graphs respectively. Statistically significance was confirmed when P values were less than 0.05. As for clinicopathologic feature analysis (including clinical data of participants and TCGA data), the Chi-square test, Student’s t-test, and nonparametric test were selected according to the corresponding application conditions. We used a Log rank test to compare survival curves of different groups and the Kaplan-Meier method to evaluate the univariate prognostic significance that was related to overall survival (OS). Multivariate prognostic analysis was performed using Cox regression analysis.

## Result

### Relative miR-9-5p expression evaluated by RT-qPCR

**Figure 1:**
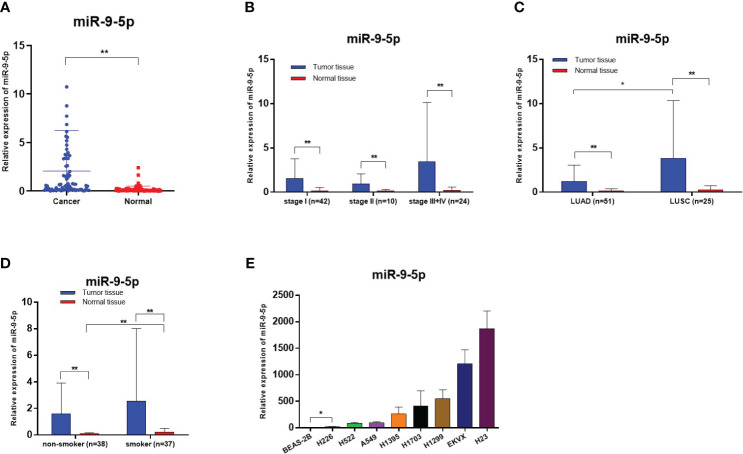
Relative miR-9-5p expression detected by RT-qPCR, *Statistical significance P < 0.05, **Statistical significance P < 0.01. **(A)** Relative miR-9-5p expression in malignant and non-malignant tissues of included patients. **(B)** Relative miR-9-5p expression in NSCLC patients of various TNM stages. **(C)** Relative miR-9-5p expression in malignant and non-malignant tissues of different histologic types. **(D)** Relative miR-9-5p expression in malignant and non-malignant tissues of different smoking states. **(E)** Relative miR-9-5p expression in BEAS-2B and various NSCLC cell lines.

The expression levels of miR-9-5p in 76 paired NSCLC tissues and adjacent noncancerous tissues were quantified by RT-qPCR. The results show that levels of miR-9-5p were significantly increased in tumor tissues, median 2.068 (25% – 75%, 0.1546–3.148) in cancer tissues, and 0.1796 (25% – 75%, 0.04113–0.1803) in adjacent noncancerous tissues. The miR-9-5p expression levels were significantly higher in lung cancer tissues (P < 0.0001) (**Figure 1A**). After dividing participants into subgroups according to various TNM stages, histological types, and smoking status, the miR-9-5p expression levels were also significantly higher in cancer tissues than in adjacent noncancerous tissues (**Figures 1B–D**). As is consistent with tissue samples’ RT-qPCR result, the relative miR-9-5p expression levels were also significantly higher in NSCLC cells than in BEAS-2B cells (**Figure 1E**).

### Clinicopathological analysis of miR-9-5p expression

We further evaluated the clinicopathological characteristics of participants that correlated to miR-9-5p expression. As for tumor tissues, the analysis results indicated that miR-9-5p expression levels were higher in relatively larger tumors (2 cm in maximum diameter as cut of value, *P* = 0.0327). Besides, miR-9-5p values were also remarkably higher in LUSC in contrast with in LUAD (*P* = 0.0143). Otherwise, there was no correlation detected between age, sex, lymphatic metastasis, pleura invasion, or vessel carcinoma embolus (**Table 1**). In addition, we further analyzed the correlation between the existence of pleural invasion or vascular cancer embolus and the miR-9-5p expression levels in tumor tissues using the Chi-square test. We used the top 50% and bottom 50% of the miR-9-5p expression levels as the cutoff value to divide the high and low miR-9-5p expression groups. We found that the high expression groups had relatively higher incidence of pleural invasion (*P* = 0.0249) and vascular cancer embolus (*P* = 0.026). As for adjacent nonmalignant tissues, the analysis results indicated that miR-9-5p expression was also increased along with tumor size (*P* = 0.0465). Interestingly, we also found that miR-9-5p was upregulated in nonmalignant tissues of smokers (*P* = 0.0099, **Figure 1D**) and males (*P* = 0.0430). In addition, the pathological subtypes information could be collected in 48 cases out of included 76 patients. Among those 48 patients, lepidic predominance accounted for 15 cases, acinar type accounted for 18 cases, papillary type accounted for 4 cases, acinar with papillary type accounted for 1 case, micropapillary type accounted for 5 cases, and solid predominant accounted for 5 cases. Furthermore, those patients were divided into three groups according to differentiated degree: well-differentiated (15 cases), moderately differentiated (23 cases), and poorly differentiated (10 cases). Among different pathological subtypes or differentiated groups, no significant difference was found regarding the expression level of miR-9-5p in tumor tissues. Otherwise, no correlation was found with other clinicopathological parameters (**Table 1**).

### Influence of miR-9-5p on the malignant phenotype of NSCLC cell lines

**Figure 2:**
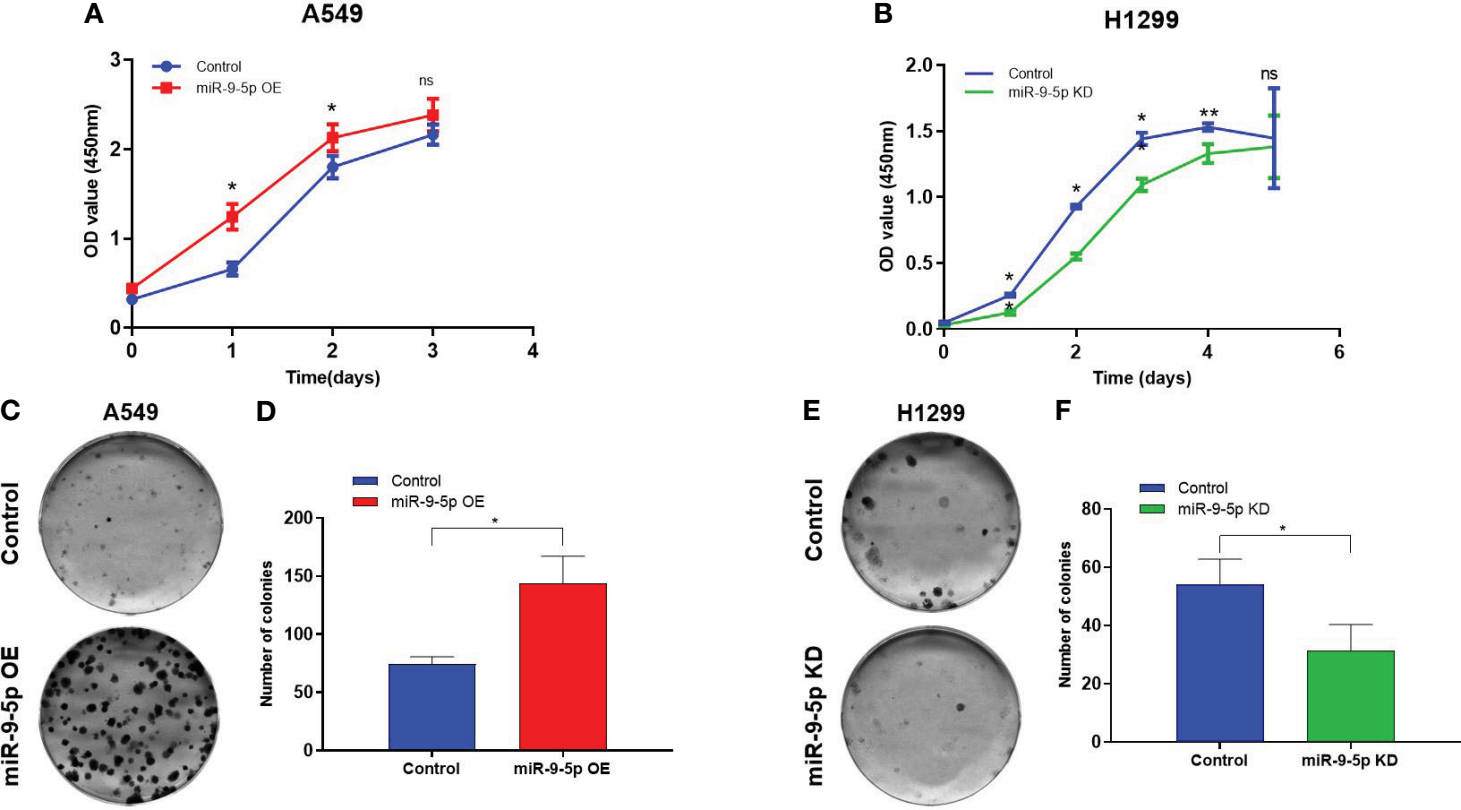
Malignant phenotype in the miR-9-5p overexpression or inhibition cell models. **(A)** Using cell growth curve assays to evaluate the proliferation capacities of A549 cells transfected with the miR-9-5p overexpression plasmid. miR-9-5p OE, cells transfected with miR-9-5p overexpression plasmid. **(B)** Using cell growth curve assay to measure the proliferation capacity of H1299 cells transfected with the miR-9-5p inhibition plasmid. miR-9-5p KD, cells transfected with miR-9-5p inhibition plasmid. **(C)** Colony formation assays result of A549 cells transfected with miR-9-5p overexpression plasmid. Remarkable differences were observed from representative pictures. **(D)** Colony numbers of A549. **(E)**. Colony formation assays result of H1299 cells transfected with miR-9-5p inhibition plasmid. Remarkable differences were observed from representative pictures. **(F)** Colony numbers of H1299. *Statistical significance P < 0.05. **Statistical significance P < 0.01. ns, no statistical significance.

The cell growth curve assay indicated that the proliferation ability of the A549 cells (miR-9-5p overexpressed) was significantly promoted in contrast with the negative control (**Figure 2A**). In addition, the proliferation of the H1299 cells (miR-9-5p inhibited) was remarkably decreased (**Figure 2B**). Consistent with cell growth curve results, the colony formation assay revealed that both colony numbers and colony sizes of A549 cells (miR-9-5p overexpressed) were significantly increased (**Figures 2C, D**). In addition, the colony numbers and colony sizes of the H1299 cells (miR-9-5p inhibited) were significantly reduced (**Figures 2E, F**).

**Figure 3:**
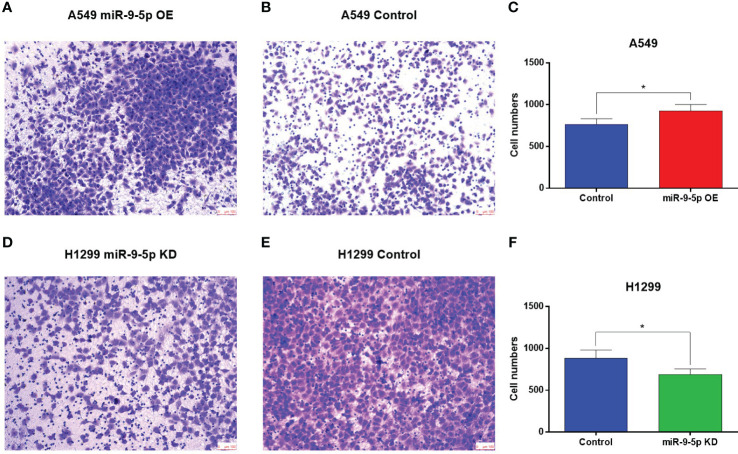
Transwell migration assay of the miR-9-5p overexpression or suppression cell models. **(A, B)** The number of migrating cells was significantly increased in the miR-9-5p overexpression group. Remarkable differences were observed from representative pictures. **(C)** The A549 cell numbers counted using Image J **(D, E)** The number of migrating cells significantly declined in the miR-9-5p suppression group. Remarkable differences were observed from representative pictures. **(F)** The H1299 cell numbers counted using Image J. *Statistical significance P < 0.05

As for migration ability, the transwell migration assay showed that the invaded cell numbers of A549 cell line transfected with miR-9-5p-OE plasmid were significantly increased (**Figures 3A–C**). Meanwhile, those of H1299 transfected with miR-9-5p-KD plasmid were significantly decreased compared with the negative control (**Figures 3D–F**).

### Bioinformatic analysis in TCGA

**Figure 4:**
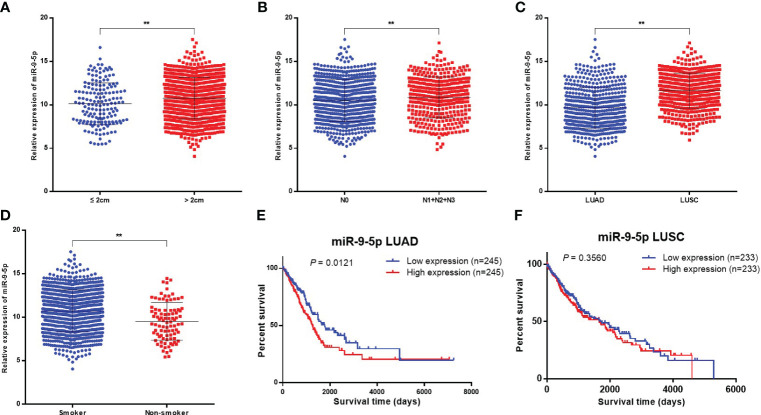
Bioinformatics analysis in TCGA database concerning the clinicopathological and prognostic significance of miR-9-5p. **(A–D)** Relative miR-9-5p expression in tumor tissues among participants of different tumor sizes **(A)**, lymphatic metastasis status **(B)**, histological types **(C)**, and smoking status **(D)**. **(E, F)** Kaplan-Meier curves for LUAD or LUSC patients stratified by miR-9-5p expression level (top 10% versus bottom 10%). ** Statistical significance P < 0.01.

Clinicopathological significance analysis of miR-9-5p in NSCLC in TCGA database was performed using Assistant for Clinical Bioinformatics (https://www.aclbi.com/). The analysis results showed that miR-9-5p expression was significantly increased along with the tumor size (**Figure 4A**, *P* = 0.0022) and the emergence of lymphatic metastasis (**Figure 4B**, *P* = 0.0141); these findings were consistent with the previous qPCR and transwell migration assay results. Moreover, miR-9-5p expression was also relatively higher in the tumor tissue of LUSC than in that of LUAD (**Figure 4C**, *P* < 0.0001). Besides, it is worth noting that we found miR-9-5p was relatively higher in the tumor tissues of smokers than nonsmokers (**Figure 4D**, *P* < 0.0001).

A total of 490 LUAD cases and 466 LUSC cases found in the TCGA database totally were included in the univariate prognostic analysis of miR-9-5p in NSCLC, patients were categorized as being in the high miR-9-5p expression group (top 10%, n = 245 for LUAD and n = 233 for LUSC) and low miR-9-5p expression group (bottom 10%, n = 245 for LUAD and n = 233 for LUSC) based on miR-9-5p expression level. Among LUAD patients, Kaplan–Meier survival analysis indicated that patients with high levels of miR-9-5p have a relatively poor prognosis (**Figure 4E**, *P* = 0.0121). In contrast with LUAD, no statistical significance could be observed among included LUSC patients in TCGA (**Figure 4F**, *P* = 0.3560).

In total, 336 LUAD cases and 382 LUSC cases in the TCGA database were included in the multivariate prognostic analysis; patients were categorized belonging to the high or low miR-9-5p expression groups (using median as the cut off value) based on their miR-9-5p expression level. Among LUAD patients, Cox regression analysis indicated that patients with high levels of miR-9-5p have a relatively poor prognosis (**Supplementary Figure 1**, *P* = 0.0368). However, no statistical significance could be observed among included LUSC patients (**Supplementary Figure 2**, *P* = 0.9616).

Using the CYBERSORT algorithm, we compared the tumor immune microenvironment of included LUAD and LUSC patients; those patients were divided into high or low expression groups according to their miR-9-5p expression level (using median as cut-off value). Among LUAD, more CD8^+^ T cells infiltration and activated dendritic cells or mast cells could be observed in high miR-9-5p expression patients, while more CD4^+^ memory T cells resting could be observed in the low expression group (**Supplementary Figures 3**, **4**). Among LUSC, more M0 macrophages could be observed in the high expression group, while more neutrophils, gamma delta T cells, and regulatory T cells (Tregs) could be observed in the low expression group (**Supplementary Figures 5**, **6**).

GO function and KEGG pathway enrichment analysis of miR-9-5p potential target genes were performed by R software package. A total of 1236 genes found by miRDB (https://mirdb.org/) were included. The top 10 messages for GO-BP, GO-CC and KEGG and the top 8 messages for GO-MF are demonstrated in the bubble graph (**Supplementary Figures 7**, **8**). GO functional analysis showed that potential target genes were mainly involved in the axon development, transport vesicle, and DNA−binding transcription factor binding (**Supplementary Figure 7**). KEGG pathway analysis showed that those potential target genes were primarily associated with the MAPK signaling pathway, endocytosis, Ras signaling pathway, and focal adhesion (**Supplementary Figure 8**).

## Discussion

In recent years, the expression changes of miR-9-5p in various kinds of tumors have been gradually reported. However, the role of miR-9-5p in lung cancer remains controversial. Gang Li et al. found that the level of miR-9-5p in NSCLC tumor tissues was higher than in normal lung tissues, and they found that miR-9-5p could promote the proliferation, migration, and invasion ability of NSCLC cell lines *in vitro*. Subsequently, it was confirmed in follow-up experiments that miR-9-5p plays a cancer-promoting role by downregulating the expression of TGFBR2 (19). However, Guodong Xu et al. also found that being transfected with miR-9-5p mimic can significantly reduce the invasion and metastasis ability of A549 and H1299 cell lines, while transfection with miR-9-5p inhibitor can enhance the migration ability and the migrating cell number (13).

In this study, through RT-qPCR detection of clinical samples, it was found that the miR-9-5p expression in cancer tissues was significantly higher than in normal lung tissues. At the same time, we also found that miR-9-5p expression in NSCLC cell lines was higher in contrast with in BEAS-2B cell lines (including H226, P = 0.0452). Moreover, the clinical information analysis of 76 included cases and the bioinformatics analysis of the TCGA database showed that the miR-9-5p expression was positively correlated with tumor diameter, suggesting that miR-9-5p might promote the proliferation of NSCLC. Subsequently, to verify this finding, we selected A549 and H1299 cell lines for *in vitro* colony formation and growth curve experiments according to the previous qPCR result. The *in vitro* experiment results showed that the proliferation of A549 was improved after overexpression of miR-9-5p, while the proliferation of H1299 was decreased after knockdown of miR-9-5p. This is consistent with the previous qPCR results of clinical samples. In addition to A549 and H1299, previous studies have also demonstrated that miR-9-5p can promote the malignant phenotypes SK-MES-1 (19), H1975 (20), and HCC827 (12) *in vitro*. This may also support the conclusion of our study to some extent. It is worth mentioning that we also found that the expression of miR-9-5p in normal lung tissues increased with the increase of tumor diameter (P = 0.0465), suggesting that the effective site of miR-9-5p may not be limited to the tumor.

As for pleural invasion and vasculature embolus, the T-test of the tumor tissue qPCR results of enrolled patients indicated that the level of miR-9-5p slightly increased in participants with pleural invasion or vasculature embolus, but the difference was not statistically significant (**Table 1**). In order to analyze the data more comprehensively, we further conducted Chi-square test analysis for this part of clinical data (using the mean expression level of miR-9-5p as the boundary value). The results suggested that the level of miR-9-5p was significantly increased in the group with pleural metastasis (P = 0.0249), and the same phenomenon also appeared in patients with vascular invasion (P = 0.026). The above information suggests that the expression of miR-9-5p is potentially related to the migration and invasion ability of NSCLC.

Meanwhile, the bioinformatics analysis also indicated that the level of miR-9-5p expression in tumor tissues was correlated with lymph node metastasis, which means the level of miR-9-5p was higher in NSCLC tumor tissues with lymph node metastases (including N1, N2, and N3) compared with cases without lymph node metastasis (N0), (*P* = 0.0141) (**Figure 4B**).

Previously, Gang Li et al. also reported that miR-9-5p expression in adjacent normal lung tissues of NSCLC patients with lymphatic metastasis was increased in contrast with that of NSCLC patients without lymphatic metastasis (19). Combined with the results of our study and previous studies on the correlation between miR-9-5p and lymphatic metastasis, we speculated that miR-9-5p may act both on lung cancer tissue and surrounding normal lung tissue and promote lymphatic metastasis. However, the qPCR results of the included 76 patients showed no significant difference in miR-9-5p levels among patients with or without lymphatic metastasis. We thought that this may be caused by the limited sample size of patients included. At the same time, to verify the influence of miR-9-5p on the invasion and migration ability of NSCLC cells under the limited clinical sample size, a transwell experiment (**Figure 3**) was performed on NSCLC cell lines (A549 for OE, H1299 for KD). The result showed that high expression levels of miR-9-5p may promote migration and invasion ability of NSCLC cells.

Interestingly, we found that among the 76 enrolled patients, the miR-9-5p expression in normal lung tissues of smokers was higher than that of non-smokers (**Figure 1D**). It is worth noting that we also found miR-182-5p and miR-183-3p were elevated in normal lung tissue of smokers previously (21). Meanwhile, the results of TCGA bioinformatics analysis showed that the miR-9-5p level in NSCLC tumor tissues of smokers was significantly higher than that of non-smokers (**Figure 4D**). The above two results suggest that the changes in miR-9-5p expression are closely related to smoking. It has been previously reported that the expression of some miRNA is significantly different between smokers and non-smokers (22, 23). Most of those miRNAs were found to be associated with lung development, lung inflammation, lung cancer, and airway epithelial differentiation (24, 25). However, A Holownia et al. reported that cigarette smoke could reduce the expression of miR-9-5p in NSCLC cell line (A549), which contradicts the conclusion of our study (7). We speculated that this may be due to the fact that A Holownia’s *in vitro* experiment of cigarette smoke on A549 was more different from the human environment, and further rigorously designed *in vitro* experiments are needed for verification. Through systematic retrieval, we found that at present, few studies have been related to the correlation between miR-9-5p and smoking behavior or tobacco smoke exposure. Therefore, our study results in this respect may be helpful to discover the correlation between smoking and miR-9-5p. It should be mentioned that SIRT1 is a potential downstream target of miR-9-5p (found by target prediction in this study), and previous studies have confirmed the target relationship between these two genes (26). It has been demonstrated previously that the activation and upregulation of SIRT1 can inhibit the proliferation of NSCLC cells and promote their apoptosis through the MAPK pathway (27), the expression of SIRT1 in NSCLC of smokers is significantly down-regulated (28). These suggests that SIRT1 may play be a tumor suppressor gene in NSCLC. Meanwhile, the expression of SIRT1 is closely related to smoking behavior, and it has also be demonstrated that the activation of SIRT1 plays a protective role in smoking-induced epithelial cell damage (29, 30). In summary, the miR-9-5p/SIRT1 pathway may be involved the process of smoking-induced lung cancer, which is worth further research to confirm.

Numerous of studies have indicated that the incidence of LUSC is closely related to smoking (31–34). Through clinical samples and TCGA database analysis we also found that the miR-9-5p expression in LUSC was significantly higher than that in LUAD. As for clinical patients, this may be due to the higher percentage of smokers (20/51 in LUAD and 17/24 in LUSC). This phenomenon further suggests that smoking behavior, miR-9-5p expression, and the occurrence and development of LUSC are potentially correlated, which is worthy of further study and exploration. At present, the mechanism with which smoking causes LUSC is not completely clear. The conclusion of our study suggests that the increased miR-9-5p level, directly or indirectly caused by smoking, may be part of the mechanism. It should be noted that the t-test of qPCR detection results in normal lung tissues suggested that the level of miR-9-5p in normal lung tissues of males was higher than that of females(*P =* 0.0430), which the authors believed might be caused by the high proportion of smokers in male patients (37/48 in males and 0/28 in females).

Moreover, survival analysis in this study showed that LUAD patients with lower miR-9-5p levels had relatively longer overall survival. This result suggests that the level of miR-9-5p expression may have certain prognostic value in LUAD patients, which also further proves the cancer-promoting role of miR-9-5p in lung cancer. It should be noted that univariate survival analysis (**Figures 4E, F**) found that the miR-9-5p expression level was not significantly correlated with the prognosis of LUSC. Further multivariable prognostic analysis of the TCGA database also indicated that the miR-9-5p expression level was not significantly correlated with the prognosis of LUSC patients either (**Supplementary Figure 2**). Therefore, we suspect that the prognosis of LUSC patients is not significant influenced by the expression level of miR-9-5p.

Immuneinfiltration analysis of tumor tissue was performed using CYBERSORT algorithm. In LUDA, the expression level of miR-9-5p was negatively correlated with CD4^+^ T cells and positively correlated with CD8^+^ T cells, dendritic cells, and mast cells. In LUSC, the level of miR-9-5p was negatively correlated with neutrophils, gamma delta T cells, and regulatory T cells and positively correlated with M0 macrophages. The immuneinfiltration analysis results indicated that miR-9-5p possibly be involved in the immune regulation of the tumor microenvironment of NSCLC, especially the T-cell-related immune response.

Finally, through potential target prediction and KEGG enrichment analysis, we found that the downstream target genes of miR-9-5p may be involved in several pathways related to the malignant phenotype of lung cancer, including cellular senescence (35), endocytosis (36), the MAPK signaling pathway (37), and the Ras signaling pathway (38). Meanwhile, it has been reported that TGFBR2 is one of the downstream target genes of miR-9-5p (19), and the KEGG analysis of this study also showed that TGFBR2 was involved in several signal pathways related to the malignant properties of lung cancer, such as cellular senescence, endocytosis, and MAPK pathways, which had been reported previously (39, 40). The above evidence further indicates that miR-9-5p is closely related to the regulatory mechanism of lung cancer.

In summary, we found that miR-9-5p is highly expressed in NSCLC tumor tissues, especially in LUSC, and miR-9-5p is associated with malignant characteristics of NSCLC. The oncogenic effect of miR-9-5p was confirmed *in vitro*. Furthermore, increased expression of miR-9-5p in normal lung tissue is closely related to smoking.

There are still some limitations to our research. First, the 76 cases included in this study were enrolled from 24 October 2018 to 20 January 2020. Due to insufficient follow-up time and limited sample size, it is not suitable for prognostic study. In order to explore the prognostic value of miR-9-5p, we evaluated the prognostic value through the bioinformatics analysis of the TCGA database. Follow-up of those participants is still being carried out, and the prognostic data of these patients will continue to be present in subsequent studies. Second, we confirmed that miR-9-5p is associated with malignant manifestations of NSCLC, and we speculate that this miRNA may also have a certain diagnostic value. Looking forward, follow-up studies should focus on peripheral blood samples and the diagnostic value of miR-9-5p.

## Conclusion

In this study we found that miR-9-5p is highly expressed in NSCLC tumor tissues, and the values are even higher in LUSC than LUAD. miR-9-5p is associated with several malignant characteristics of NSCLC such as tumor size, lymphatic metastasis, pleural invasion, and vascular thrombus; *in vitro*, miR-9-5p can promote proliferation, migration, and invasion of NSCLC cells. Meanwhile, in normal lung tissue, increased miR-9-5p levels are closely related to smoking. In terms of prognosis, increased level of miR-9-5p expression in LUAD was associated with poor prognosis. Our study can provide support for the finding of potential prognostic or therapeutic targets for NSCLC patients and the exploration of the relationship between smoking and lung cancer.

## Data availability statement

The raw data supporting the conclusions of this article will be made available by the authors, without undue reservation.

## Ethics statement

The studies involving humans were approved by Ethics Committee of Beijing Chest Hospital. The studies were conducted in accordance with the local legislation and institutional requirements. The participants provided their written informed consent to participate in this study. Written informed consent was obtained from the individual(s) for the publication of any potentially identifiable images or data included in this article.

## Author contributions

TZ: Investigation, Resources, Software, Supervision, Writing – original draft, Writing – review & editing. XD: Funding acquisition, Resources, Supervision, Writing – review & editing. YC: Conceptualization, Funding acquisition, Resources, Supervision, Writing – review & editing. YZ: Data curation, Formal analysis, Methodology, Writing – review & editing. MG: Methodology, Writing – review & editing. ZW: Data curation, Resources, Writing – review & editing. WL: Conceptualization, Methodology, Software, Supervision, Writing – original draft, Writing – review & editing.
