# Supplementary figures and images for: Clinical significance of miR-9-5p in NSCLC and its relationship with smoking
Source: Front Oncol. 2024 Apr 2;14:1376502. doi: 10.3389/fonc.2024.1376502 (PMC11018953; doi:10.3389/fonc.2024.1376502)

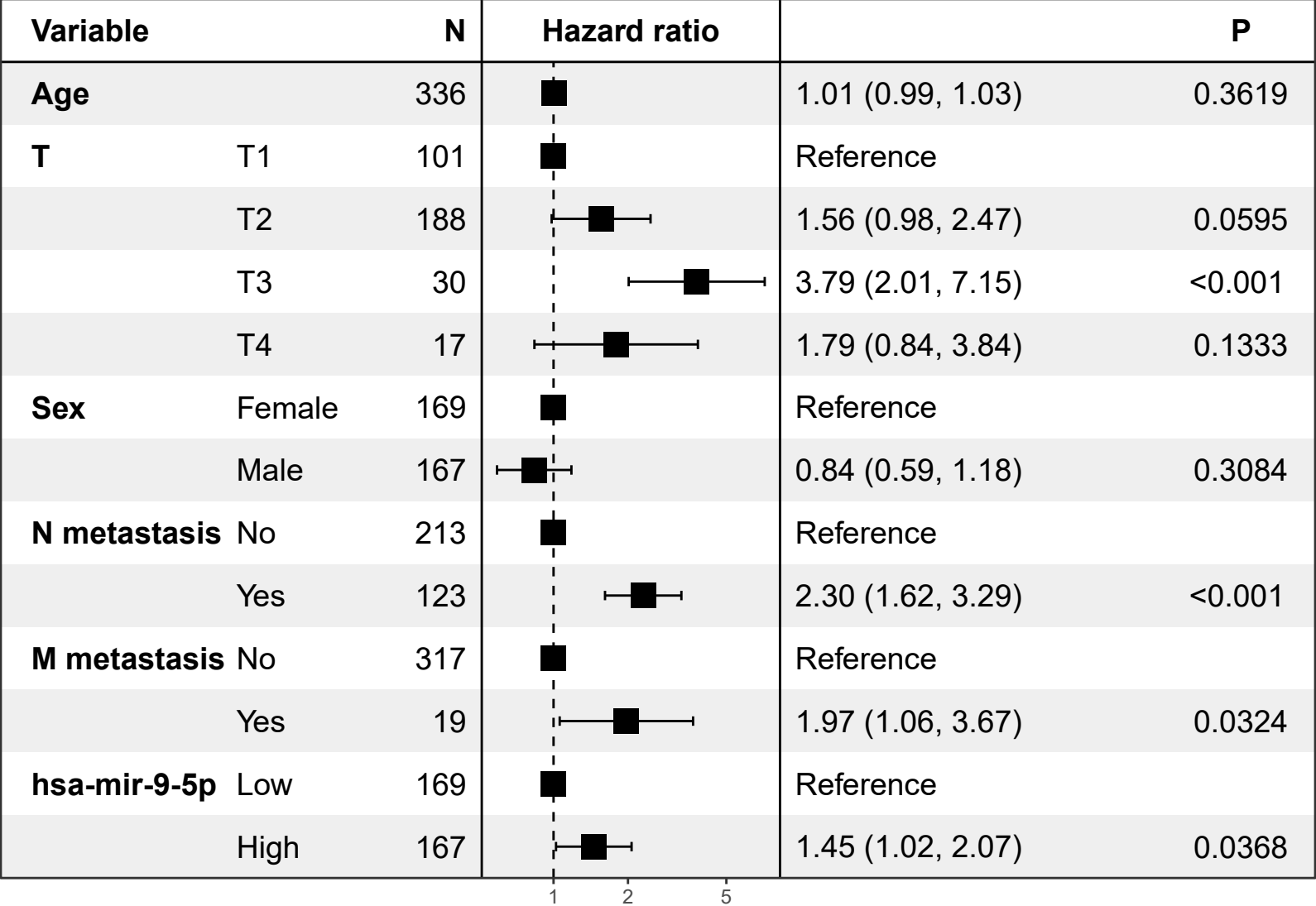

Supplement: Supplementary file 1 [file Image_1.pdf]

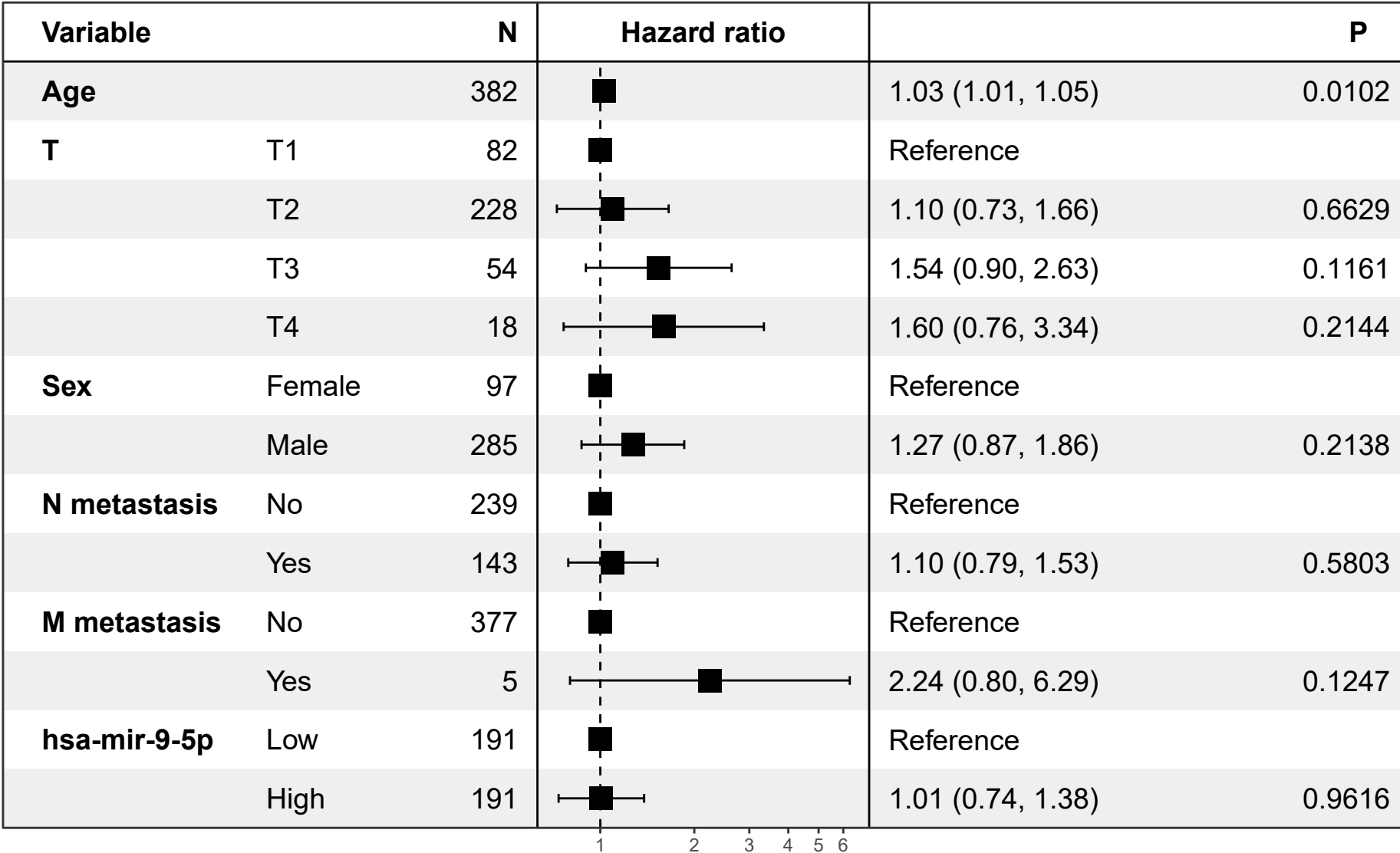

Supplement: Supplementary file 2 [file Image_2.pdf]

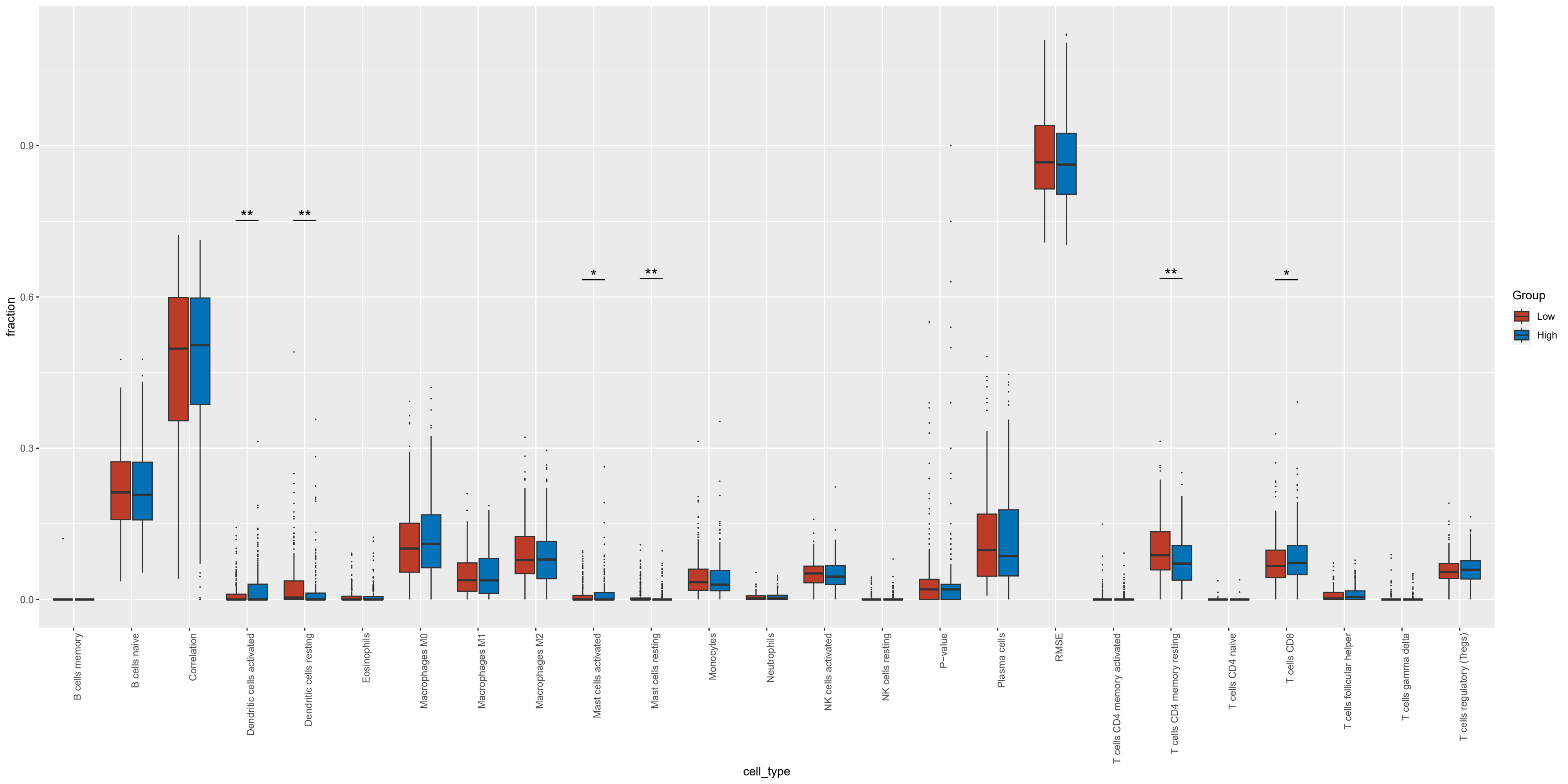

Supplement: Supplementary file 3 [file Image_3.pdf]

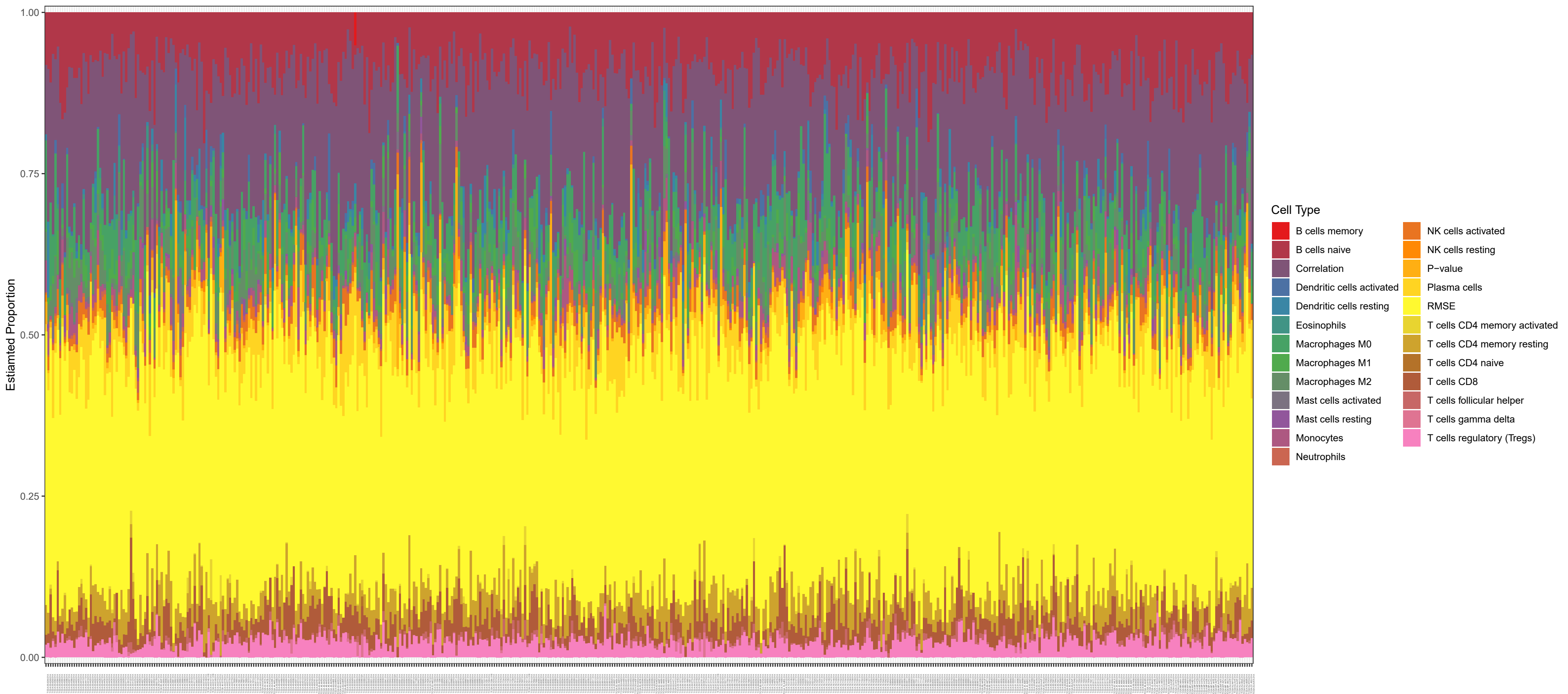

Supplement: Supplementary file 4 [file Image_4.pdf]

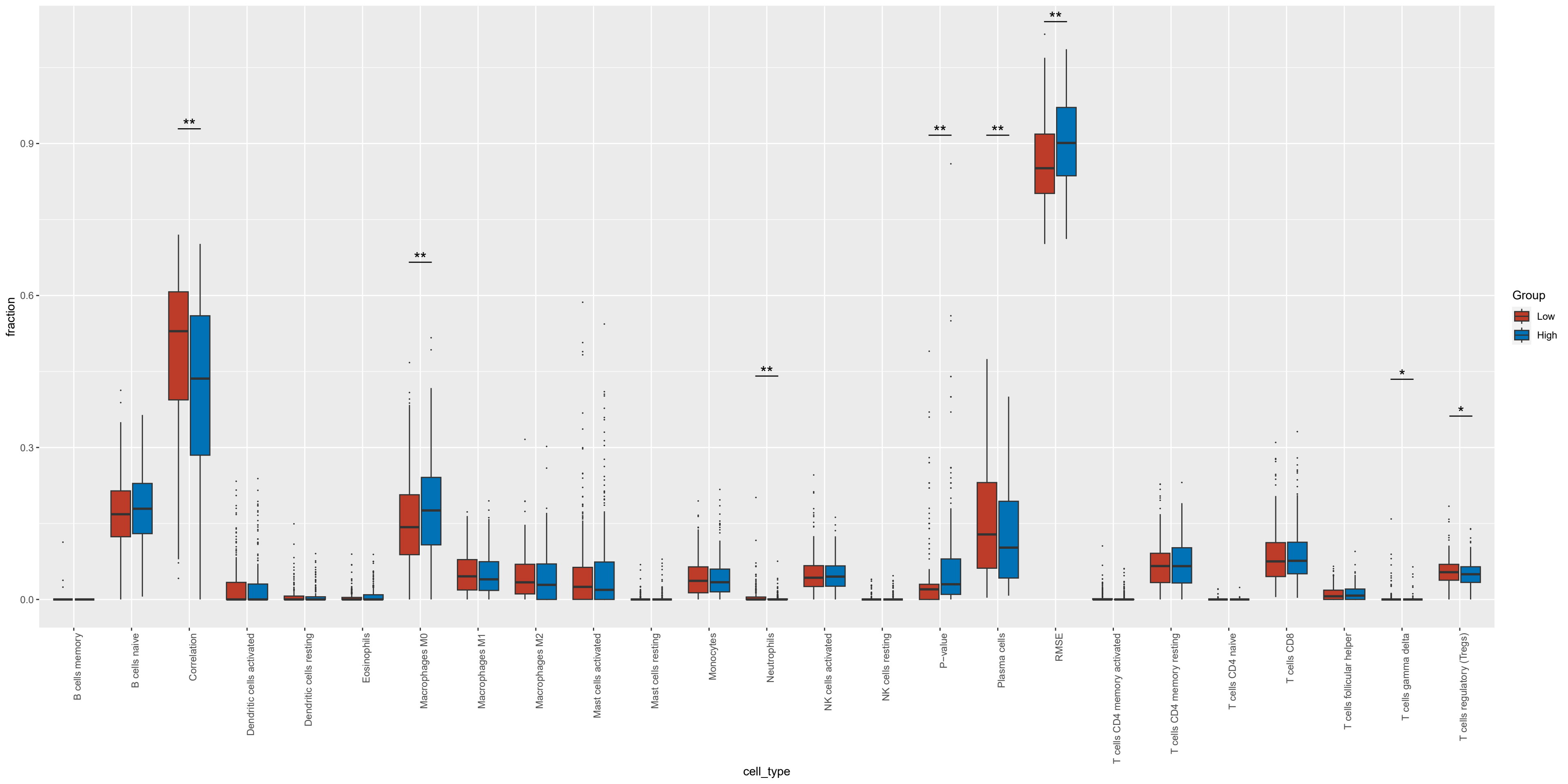

Supplement: Supplementary file 5 [file Image_5.pdf]

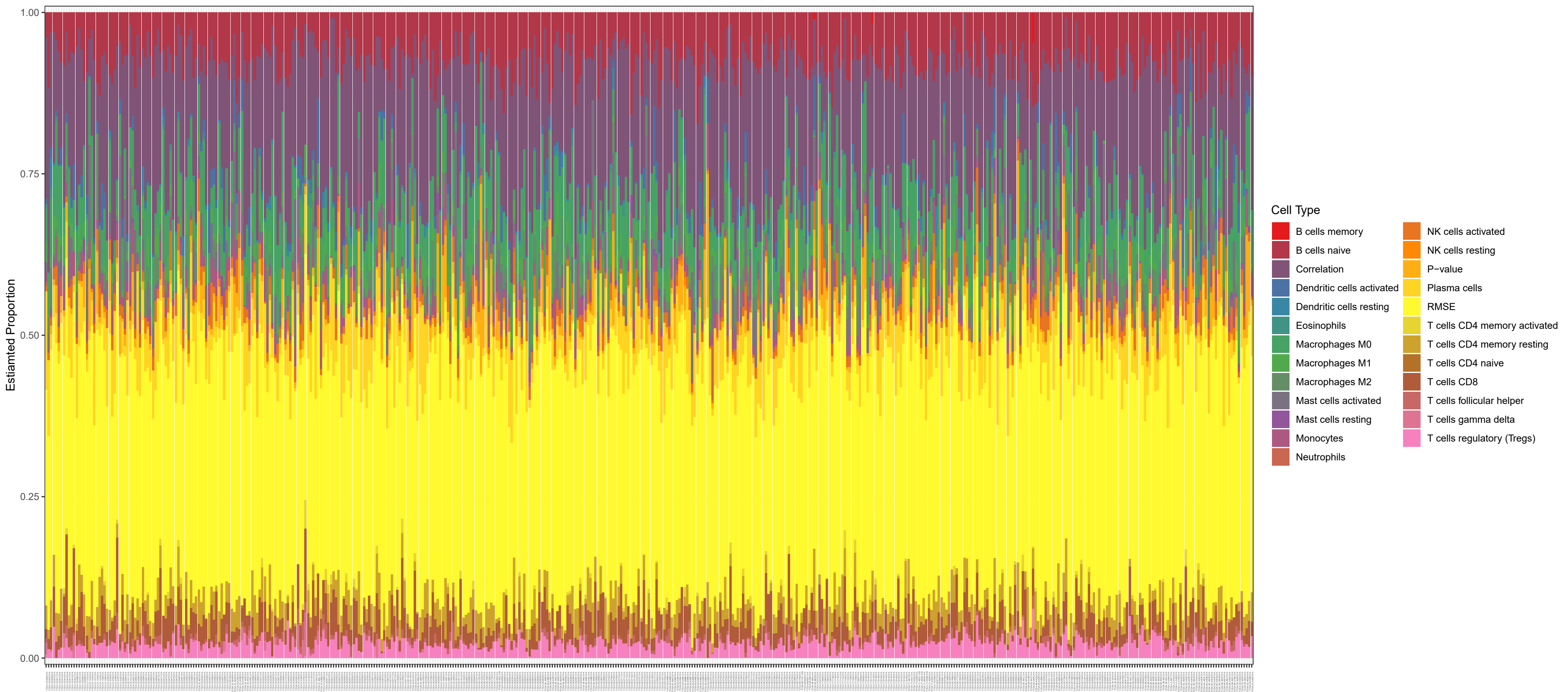

Supplement: Supplementary file 6 [file Image_6.pdf]

# GO Enrichment

GeneRatio

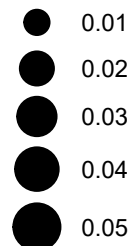

p.adjust

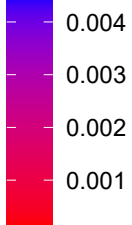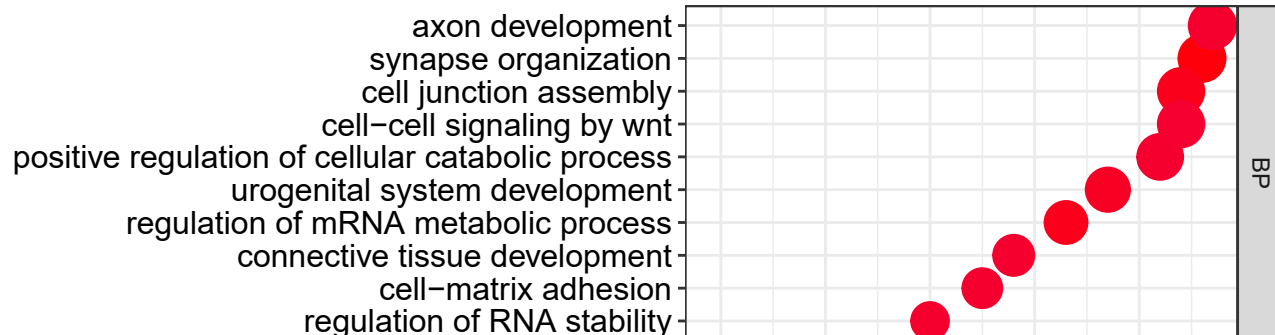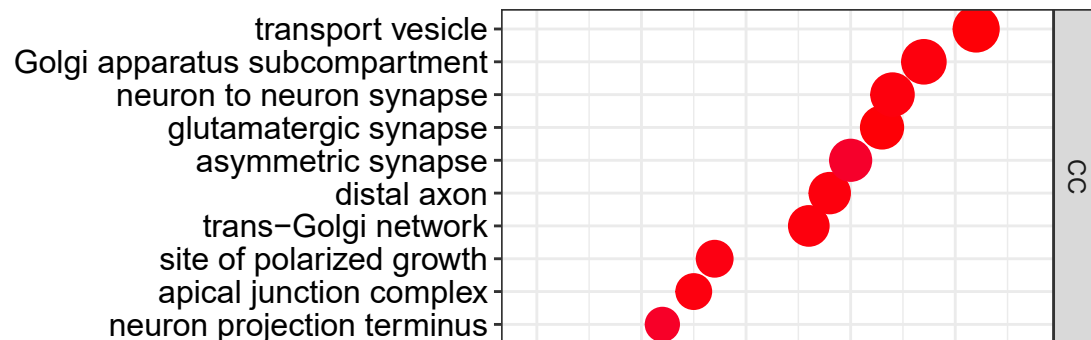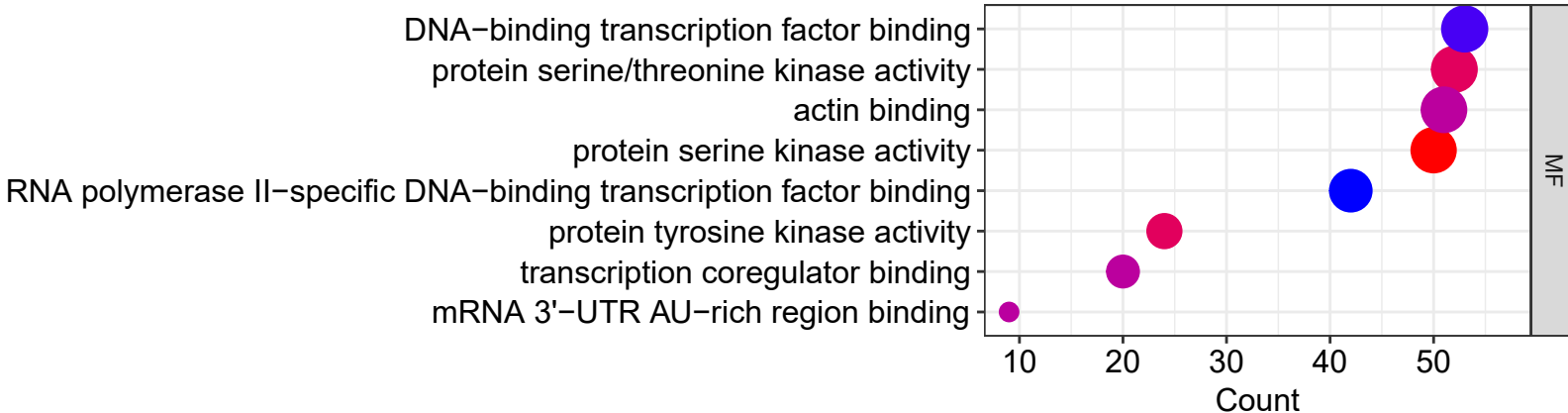

Supplement: Supplementary file 7 [file Image_7.pdf]

# Enrichment KEGG\_dot

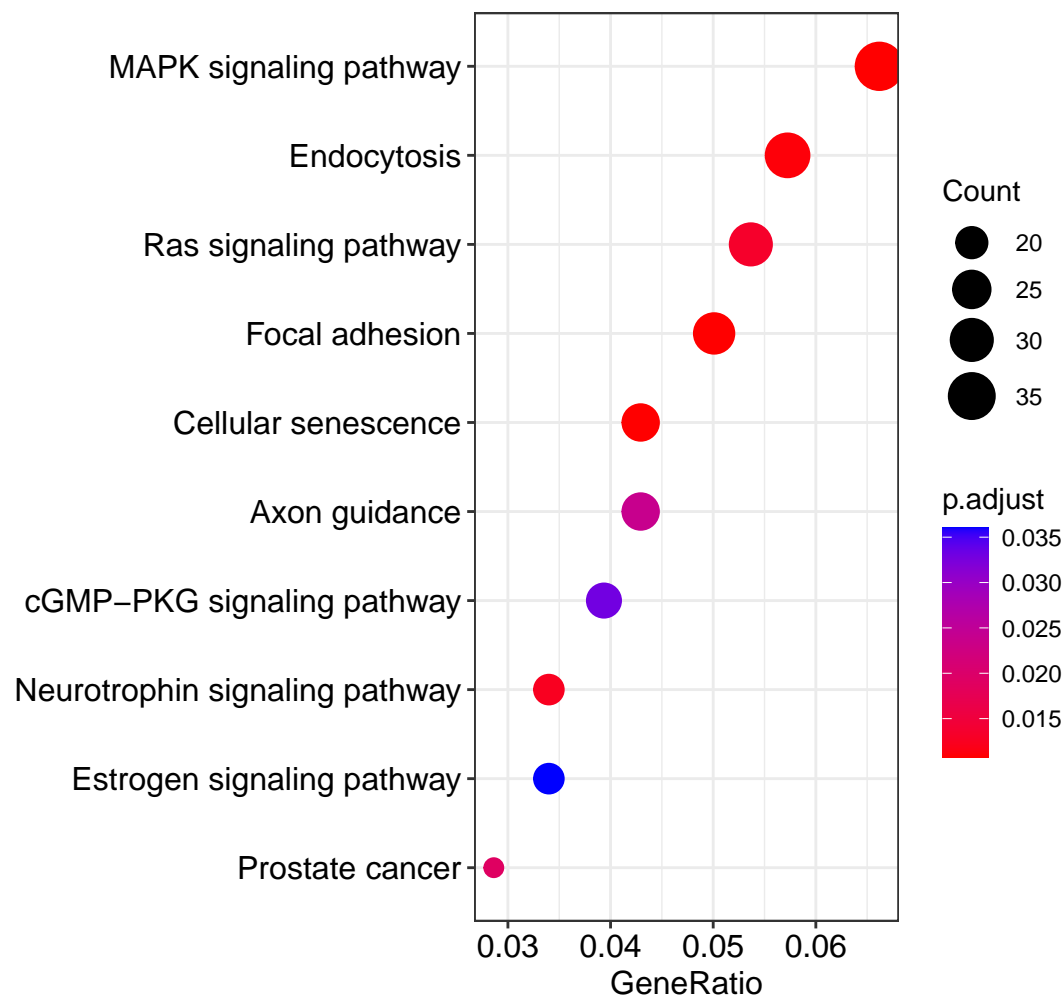

Supplement: Supplementary file 8 [file Image_8.pdf]
